# Application of Deep Multi-Scale Representation Learning Based on Eye-Tracking and Facial Expression Data in Cognitive Decline Assessment

**DOI:** 10.3390/s26092600

**Published:** 2026-04-23

**Authors:** Yanfeng Xue, Xianpeng Luo, Shuai Guo, Tao Song

**Affiliations:** 1 Shanghai Key Laboratory of Intelligent Manufacturing and Robotics, School of Mechatronic Engineering and Automation, Shanghai University, Shanghai 200444, China; kuailede@shu.edu.cn (Y.X.); luoxianpengjiayou@163.com (X.L.); 2 National Demonstration Center for Experiment Engineering Training Education, Shanghai University, Shanghai 200444, China; guoshuai@i.shu.edu.cn; 3 School of Mechatronic Engineering and Automation, Shanghai University, Shanghai 200444, China

**Keywords:** eye-tracking, facial expression data, multimodal digital biomarkers, deep learning, cognitive decline

## Abstract

Digital biomarkers derived from eye-tracking and facial expression hold significant potential for the non-invasive screening of cognitive decline (CD). However, existing approaches predominantly rely on single-task or feature engineering-based unimodal methods, which struggle to capture complex temporal behavioral patterns. While deep learning (DL) excels at extracting hierarchical features and intricate temporal dynamics from behavioral sequences, its application in this specific multimodal sensing domain remains exploratory. Addressing this gap, this study designed an assessment system integrating five multi-dimensional cognitive paradigms and collected eye-tracking and facial expression data from 20 healthy controls (HC) and 20 individuals with CD. For these multimodal sequences, we propose a deep neural network capable of multi-scale representation learning. By utilizing subspace exploration and multi-scale convolutions, this architecture extracts deep representations directly from data and incorporates a decision fusion mechanism to enhance diagnostic robustness. Experimental results demonstrate that our method achieves a 90% classification accuracy, outperforming machine learning models. Furthermore, statistical analyses conducted in this study validated several features associated with CD and also explored some novel potential behavioral patterns. This study confirms the feasibility of a DL framework based on eye-tracking and facial expression signals for identifying CD, providing a reference for developing objective and efficient digital screening tools.

## 1. Introduction

Against the backdrop of intensifying global aging, the prevalence of dementia continues to escalate [1]. According to the Seventh National Population Census, the number of dementia patients in China alone has reached approximately 15.07 million [2]. AD, the most common subtype of dementia [3], imposes a substantial societal burden due to its irreversible nature and the consequent cognitive decline [4]. Given the current absence of curative treatments, identification and intervention during the prodromal stage of Mild Cognitive Impairment (MCI) are considered crucial for delaying disease progression [5,6]. However, existing screening methods primarily rely on neuroimaging techniques (e.g., MRI, PET), which are constrained by high costs and strict equipment requirements, or clinical cognitive scales (e.g., MMSE, MoCA), which are often limited by subjectivity and low sensitivity [7,8]. These methods lack sufficient sensitivity for early detection and are difficult to deploy on a large scale. Consequently, there is an urgent need to develop novel, objective, and efficient tools for early cognitive screening.

In recent years, detection methods based on Digital Biomarkers have garnered significant attention due to their non-invasiveness, real-time capabilities, and cost effectiveness [9,10,11,12]. Among these, eye movements and facial expressions have emerged as critical behavioral modalities for detecting cognitive decline.

Eye tracking is a real-time sensing technology that quantifies neurological deviations by monitoring and analyzing ocular movement patterns such as fixation, saccades, and pupil responses [13,14]. Eye movement abnormalities can be detected using various experimental tasks. By assessing visual exploration behavior in a clock reading task, Mosimann et al. [15] demonstrated that AD patients exhibited a reduced percentage of fixations within regions of interest (ROI), delayed first fixations, and decreased saccadic amplitudes. Such abnormal visual exploration patterns may be associated with parietal dysfunction or may arise from a functional imbalance between the impaired occipitoparietal pathway and the relatively preserved occipitotemporal visual network. Regarding pupillary dynamics, Parra et al. [16] employed the Short-Term Memory Binding Task (STMBT) and found that abnormal pupil dilation in AD patients correlates with declined STMBT performance; notably, MCI patients also manifested impaired exploratory eye movements accompanied by STMBT deficits, suggesting a risk of conversion to dementia. Furthermore, the Pupillary Light Reflex (PLR) task, which measures dynamic pupil changes under light stimulation, serves as a rapid and effective screening tool; AD patients typically exhibit prolonged constriction latency and reduced amplitude/velocity, which may reflect potential parasympathetic dysfunction [17]. The saccade task is an effective screening paradigm for CD. Typically, two types of tasks are used: the pro-saccade task (PS) and the anti-saccade task (AS). Studies indicate that metrics such as latency, velocity, and amplitude are impaired in patients with CD [18]. In a PS task, Yang et al. [19] found that saccadic latency was prolonged in patients with aMCI and mild-to-moderate AD, which correlated with decreased MMSE scores. Pereira et al. [20] discovered that during the AS task, the latency and frequency of eye movements were significantly altered in patients with CD. Compared to the HC group, the AD group exhibited the most severe decline in executive function, while the MCI group demonstrated an intermediate performance. These metrics can sensitively reflect cognitive deficits in inhibitory control and working memory, both of which involve the dorsolateral prefrontal cortex [21]. Concurrently, facial expression features, analyzed through macro-expressions, micro-expressions, and emotion recognition capabilities, reflect the degradation of potential cognitive and social functions [22,23]. Jiang et al. [24] reported that during emotion-eliciting events, dementia patients display significantly fewer positive expressions and increased negative expressions. Similar studies have observed abnormal zygomaticus activity (the muscle controlling smiling) in dementia patients compared to HCs [25].

Eye-tracking and facial data constitute high-dimensional behavioral sequences reflecting cognitive states. Machine learning (ML) methods in artificial intelligence are crucial for deciphering these complex signals. In visual search tasks, ML can effectively identify MCI subjects with dementia-like eye movement patterns when distinguishing previously seen target stimuli from distractors [26]. In emotion expression tasks, ML classification models built upon Facial Action Units (FAU) play a key role in objectively discriminating emotional states like depression and anxiety within MCI populations [27]. Although ML has been widely applied to single cognitive function tasks, recent research has gradually shifted towards integrated, multi-modal composite task paradigms. Zhang et al. [28] con-structed a multi-task paradigm based on drawing, gait, and eye movements, developing feature-based Light-GBM models for each task and their combinations. The final model, which fused eye-movement and drawing data, achieved optimal performance, significantly outperforming single-task models.

Nevertheless, the introduction of Deep Learning (DL) has provided new perspectives in this field. Unlike feature-based ML methods, DL can directly extract hierarchical features from raw data, granting it a significant advantage in capturing subtle and complex temporal patterns within behavioral sequences. However, current DL methods for eye-tracking and facial expression data remain in the exploration phase for AD detection. Considering that these sequences inherently possess temporal dependencies and local correlations, drawing upon time-series modeling methods from other fields may be a feasible approach. Convolutional Neural Networks (CNN) demonstrate significant advantages in temporal feature extraction, effectively capturing local dependencies and contextual information within data, and have been widely applied in surface electromyography (sEMG) signal analysis [29] and text classification tasks [30]. Given that Eye movement and facial expression sequences also possess comparable temporal structures, therefore, applying CNN to analyze these data is a direction worth exploring. 

Parallel to the continuous evolution of algorithms, the choice of assessment environment has increasingly gained prominence. Traditional laboratory assessments impose strict requirements on physical venues and equipment, thereby limiting their applicability. In contrast, Virtual Reality (VR) technology has emerged as a promising alternative due to its controllable virtual scenarios, portability, and cost-effectiveness [31,32]. Xu et al. [33] developed a VR-based cognitive assessment tool integrated with eye tracking, demonstrating a high correlation between their Support Vector Regression model and MoCA scores. Similarly, Mannan et al. [34] utilized an immersive supermarket task to prove that spatiotemporal behavioral features captured within a VR environment can effectively differentiate heterogeneous cohorts. These studies indicate that VR-based cognitive behavioral analysis is an effective assessment modality, holding great promise for large-scale cognitive impairment screening in the future.

In summary, digital biomarkers based on eye-tracking and facial features are effective tools for AD identification. However, existing methods primarily focus on single-task or feature-based ML approaches, while research on DL remains relatively limited. Furthermore, the assessment environments and equipment they rely upon suffer from numerous limitations, making it difficult to meet the demands of large-scale screening. To this end, this study utilized VR technology to develop five multidimensional cognitive assessment paradigms, systematically collecting eye-tracking and facial expression data from 40 participants. Building upon this, we proposed a Multi-Task Multi-Stream Parallel Convolutional Neural Network (MT-MSPCNN). This architecture employs a subspace exploration strategy and multi-scale convolutional streams to learn hierarchical representations directly from the data, while enhancing classification robustness through a decision fusion mechanism. This method achieved a 90% recognition accuracy in distinguishing between the HC and CD groups. Additionally, this study statistically analyzes multiple features and explores several potential behavioral indicators, offering new insights into behavioral changes in early-stage AD.

## 2. Materials and Methods

### 2.1. Hardware and Data Acquisition System

**Figure 1:**
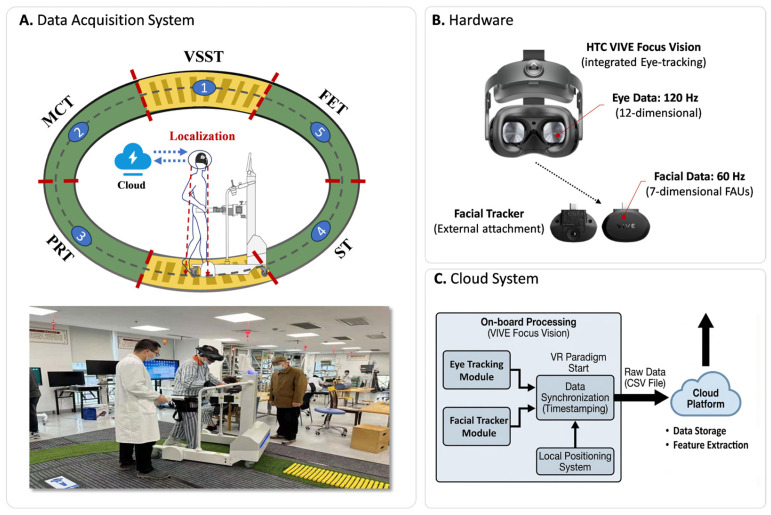
Hardware and multimodal cognitive assessment system.

All experiments were conducted in a dedicated low-reverberation room to minimize auditory distractions for participants. A floor mat was placed at the center of the experimental area, marked with five trigger positions corresponding to the VR paradigms, as shown in Figure 1.

The subject wore the HTC VIVE Focus Vision (hereinafter referred to as Focus) and moved to the designated location under the guidance of an assistive walking robot (iReGo). Once Focus’s positioning system confirms the subject has arrived at the correct location, the system will automatically initiate the corresponding VR paradigm. It is important to note that the iReGo was used solely for guidance during walking and did not participate in any data acquisition. To address any unexpected situations, a professional physician supervised the entire experiment, providing intervention and assistance when necessary. Upon completion, Focus saved the collected data into a CSV file, which was subsequently uploaded to a cloud server. All subsequent feature extraction and computational analyses were performed on the cloud platform.

### 2.2. Biosignal Extraction Based on Sensors

**Figure 2:**
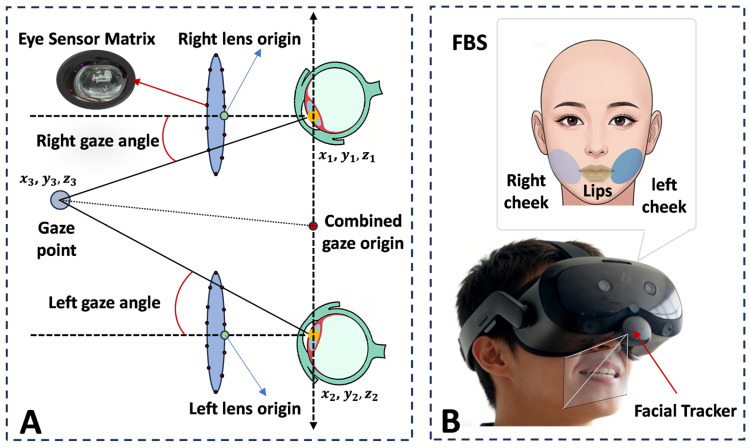
Acquisition of multimodal digital biosignals. (**A**) Built-in eye-tracking sensors quantify oculomotor dynamics. (**B**) The facial tracker captures facial action parameters.

To capture the dynamic behavioral responses of the subjects during cognitive tasks, this study utilized the built-in eye-tracking sensors of the Focus headset and an externally attached HTC Facial Tracker (hereafter referred to as FT) to acquire the subjects’ oculomotor and facial time-series signals (Supported by the VIVE SDK), as illustrated in Figure 2.

For the oculomotor signals, data were acquired at a sampling rate of 120 Hz. In terms of the sensing mechanism, the Focus headset locates the pupil center by capturing corneal reflections, thereby estimating the rotation angle and gaze direction of the eyes (Figure 2A). To ensure the accuracy of this mechanism, all subjects were required to complete a standard five-point eye-tracking calibration prior to the formal experiment. Based on this, we extracted 6 dimensions for the 3D origin coordinates of both eyes, 3 dimensions for the combined gaze point coordinates, and 2 dimensions for the physical pupil sizes of both eyes, for a total of 11 oculomotor dimensions for analysis.

Regarding the facial signals, the FT module utilizes a monochrome camera to capture the muscular movement trajectories of the subject’s lower jaw region in real time at a sampling rate of 60 Hz. The system directly maps these facial dynamics into 38 Facial Blend Shapes (FBS), as shown in Figure 2B. To quantify the subjects’ facial expressions, this study selected 6 dimensions of FBSs, specifically including: 2 dimensions describing lip opening, 2 dimensions describing smile and sadness of the left cheek, and 2 dimensions describing happiness and sadness of the right cheek. The activation intensity of each dimension was normalized to a continuous scale ranging from 0 to 1. In this study, we set the system’s update frequency to 120 Hz. Under this configuration, the eye-tracking data (120 Hz) synchronized with the frame rate, while the facial expression data (60 Hz) was up-sampled to 120 Hz by nearest-neighbor interpolation to maintain alignment.

### 2.3. Experimental Setup

**Figure 3:**
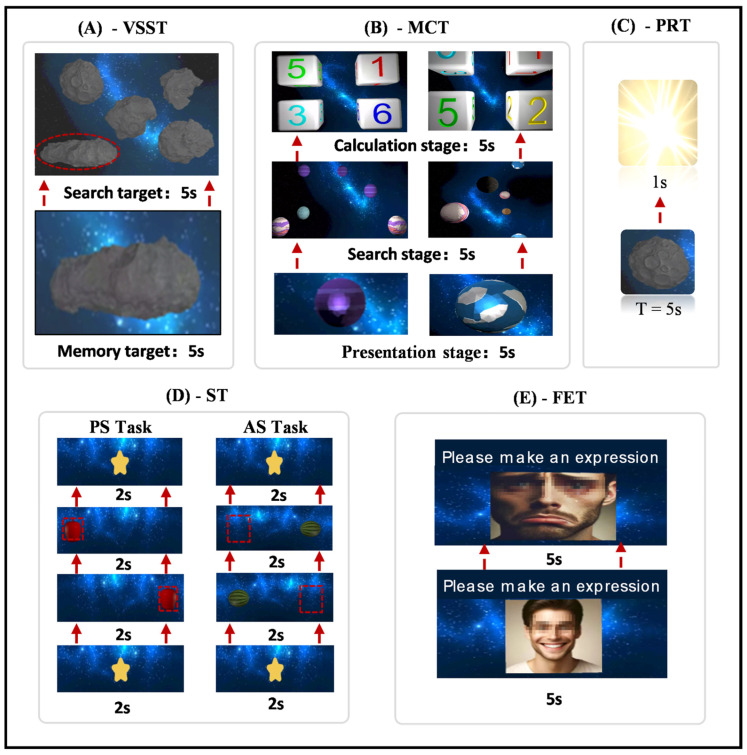
Flowchart of the multidimensional cognitive assessment paradigm. (**A**) Visual Short-term memory and Search Task. (**B**) Memory Calculation Task. (**C**) Pupillary Response Task. (**D**) Saccade Task. (**E**) Facial Emotion expression Task.

Based on the above research, this cognitive screening protocol comprised five gamified experimental paradigms, all developed in Unity. Participants were required to complete the following tasks sequentially: the Visual Short-term memory and Search Task (VSST), the Memory Calculation Task (MCT), the Pupillary Response Task (PRT), the Saccade Task (ST), and the Facial Emotion expression Task (FET). The overall procedure is illustrated in Figure 3.

Data collection occurs only within the time interval from the start to the end of each paradigm. For the first four tasks, only eye-tracking data is collected, with facial expression data filled with zero values. The emotion expression task simultaneously collects both eye-tracking and facial expression data. Additionally, the system records the name of the current paradigm in real time to enable accurate identification during subsequent data processing. The details of the paradigm are described below.

VSST: This task consisted of two phases: memorization and search. First, participants were required to memorize a static three-dimensional target object presented for 3 s. Subsequently, they had to search for and maintain fixation on this target among five subsequently displayed static objects within an 8-s time limit. This task consists of only a single trial.

MCT: Unlike the static computational task in Reference [33], this study explores participants’ performance in integrating short-term memory with dynamic search tasks. This task comprised three phases: presentation, search, and calculation. In the presentation phase, participants memorized a 3D target with a specific shape and color for 3 s. Then, within a 10-s period, they had to count the appearances of this target among a dynamically moving group of objects. Finally, in the calculation phase, based on verbal instruction, participants had to fixate on the correct numerical value from four static number options within 5 s. This task was performed in two trials, each comprising all three phases.

PRT: Subjects must maintain fixation on a target object for 5 s. This phase aims to stabilize the subject’s visual attention. During the final stage of target presentation, a 1-s light stimulus (a bulb effect, implemented in Unity) with a luminance of 24.6 cd/m^2^ (parameters based on reference [35]) was presented. This task was repeated for two trials.

ST: Participants first fixated on a central target for 2 s. Subsequently, the central target disappeared, and a new target was presented on either the left or right side for 2 s. The distance between the new target and the central target was 0.7 m. Participants performed both the PS and AS tasks. The PS task consisted of 6 trials requiring participants to direct their gaze toward the new peripheral target, whereas the AS task consisted of 6 trials requiring them to look in the opposite direction.

FET: This task required participants to voluntarily express two emotions, “happy” and “sad,” each for a duration of 5 s, following verbal instruction. To assist participants in uniformly expressing emotions, a corresponding emotional portrait was displayed synchronously during task execution for reference. Each emotion was performed only once.

Furthermore, prior to the experiment, the headset’s locking mechanism was firmly adjusted for each participant to prevent any relative displacement between Focus’s sensors and the subject’s head. During the task, participants performed the experiment in a confined space as instructed to minimize motion artifacts caused by head movement during data collection.

### 2.4. Cohort

Participants were recruited from Shanghai Tongji Hospital, Tongji University; exclusion criteria included fundus diseases and facial paralysis. All individuals underwent MoCA-B assessment by a neurologist. Group classification (HC, AD, MCI) was determined by combining education history with MoCA-B performance. For participants with less than 6 years of education, classifications were defined as AD (<13), MCI (14–19), and HCs (20–30). For those with 7–12 years of schooling, the criteria were <15 (AD), 16–22 (MCI), and 23–30 (HC). Individuals with more than 12 years of education were categorized as AD if scoring < 16, MCI if scoring 17–24, and HCs if scoring 25–30.

Given the limited sample size of AD and MCI participants in this study, to enhance the robustness of statistical analyses, the AD and MCI groups were combined into a single CD group. Consequently, the final cohort comprised 40 participants: 20 in the HC group and 20 in the CD group (15 MCI, 5 AD). Demographic and baseline characteristics of the participants are summarized in Table 1. The Mann–Whitney U test was employed for statistical analysis. The study was conducted in accordance with the Declaration of Helsinki, and the protocol was approved by the Ethics Committee of Shanghai University (ECSHU 2023-075). Written informed consent was obtained from all participants or their legal guardians after a full explanation of the study’s purpose and procedures.

### 2.5. Data Analysis

#### 2.5.1. Data Preprocessing

First, to address the common blink artifacts in eye-tracking signals, we utilize Focus’s built-in binocular aperture module (with values ranging from 0 to 1) to establish a blink detection mechanism. Specifically, we set an empirical threshold of τ=0.9. When the opening value $O_{t}<\tau$, the data is marked as a blink and discarded. To restore the continuity of the time series, the missing segments are filled in using linear interpolation. Subsequently, to effectively attenuate high-frequency noise in pupil diameter caused by environmental or device jitter while preserving information sensitive to cognitive load, we adopt a 2nd order Butterworth filter based on the method described in [36]. Let $x_{\left(t\right)}$ denote the original pupil diameter sequence, ${\hat{x}}_{n}^{s}\left(t\right)$ denote the filtered smoothed sequence. The time-domain recursive form of this filtering process is defined as follows:

(1) $${\hat{x}}_{n}^{s}\left(t\right)=\frac{1}{\Omega }\left(\sum_{i=0}^{p}h_{i}x\left(n-i\right)-\sum_{j=1}^{q}g_{j}\hat{x}\left(n-j\right)\right)$$

where Ω is the normalization coefficient and $h_{i}$ and $g_{j}$ are filter coefficients, with the order set to *p* = *q* = 2. The cutoff frequency is set to 1/8 of the sampling rate to ensure that the dynamic characteristics of the pupil light reflex are preserved while eliminating noise. After completing signal denoising, we calculate the endpoint coordinates of the merged line of sight based on the origin and direction vector of the binocular line of sight, and compute its instantaneous velocity.

**Figure 4:**
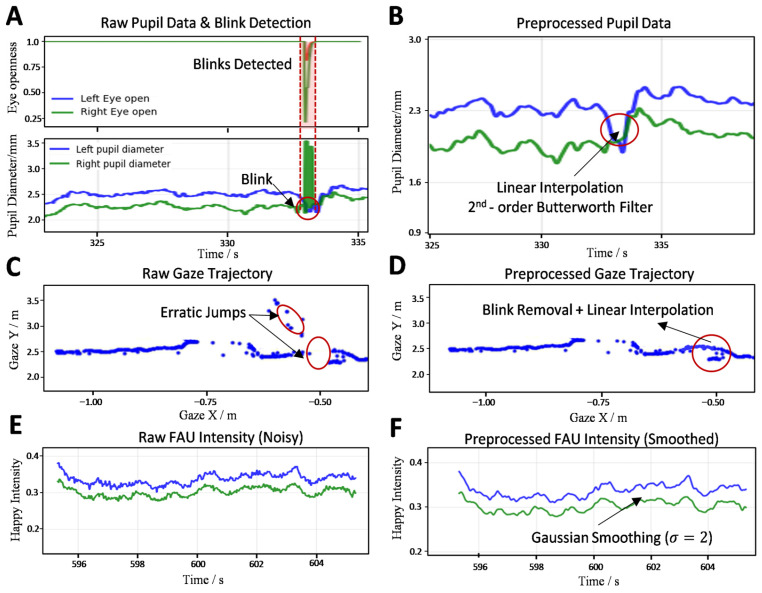
Overview of the Multi-modal Data Preprocessing Pipeline and Results. (**A**,**C**,**E**) Raw data showing artifacts from blinks and sensor noise. (**B**,**D**,**F**) Preprocessed data after applying blink detection, interpolation, and filtering techniques.

Based on this, the I-VDT algorithm [37] was employed to identify fixations and saccades using the following parameters: v = 30°/s, time = 130 ms, dispersion threshold = 2°, and minimum saccade duration = 0.04 s. This algorithm segments continuous eye-tracking sequences into fixations and saccades, providing a foundation for feature extraction in Section 2.5.2. Finally, for facial expression data, to suppress random jitter noise during facial landmark capture, we applied Gaussian filtering (σ = 2) to smooth the facial expression sequences. The results of data preprocessing based on the method are shown in Figure 4.

#### 2.5.2. Eye and Facial Feature Extraction

To compare the performance of DL models, we extracted 27-dimensional features from the preprocessed data. Among these, all metrics in the VSST, MCT, and ST were derived from the merged 3D gaze coordinates of both eyes; features in the PRT were extracted solely from the pupil sizes of both eyes; and features in the FET were computed based on the 4-dimensional emotion intensity values output from the left and right cheeks. These features reflect cognitive functions relevant to different tasks and were used for subsequent ML model training. Feature details are shown in Table 2.

VSST (5-dim): To quantify visual search efficiency and stability, we defined ROIs for the target object within the candidate cluster. Following the methodology in [15], four oculomotor metrics were extracted: ROI fixation percentage (${Fix}_{ROI}$), time to first fixation on ROI ($T_{fROI}$), total dwell time on ROI ($T_{ROI}$), and saccade length ($S_{l}$). Furthermore, to robustly characterize the stability of gaze fixation on the target, we introduced the Median ROI Fixation Time ($T_{mROI}$). This metric is defined as the median duration of all fixation points falling within the ROI during the search phase.

PRT (6-dim): To assess the dynamic pupillary light reflex, we first calculated the baseline pupil diameter. Following [36], the baseline $\mu_{T_{b}}$ was computed as a running mean over the pre-stimulus period $T_{b}$, updated iteratively as:

(2) $$\mu_{T_{b}}=k/\left(k+1\right)\mu_{T_{b}}+1/\left(k+1\right){\hat{x}}_{n}^{s}\left(t\right)$$

where ${\hat{x}}_{n}^{s}\left(t\right)$ is the pre-processed pupil diameter and k represents the sample index incrementing over $t\in \left[0,T_{b}\right]$. Subsequently, following the method in [38], six pupillary features were calculated for each eye during the light stimulation phase: maximum change acceleration (${AC}_{max}$), maximum change velocity (${VC}_{max}$), diameter range ($R_{d}$), response latency ($T_{1}$), time to maximum velocity ($T_{2}$), and time to maximum diameter change ($T_{3}$). The final value for each feature was the average of the results from the left and right eyes. The task was repeated twice, and the value used for final analysis for each feature was the average across the two rounds.

FET (6-dim): To quantify emotional expressiveness, we extracted intensity (I), duration (T), and response latency (L) for both “Happy” and “Sad” conditions. Intensity ($I_{happy},I_{sad}$) is defined as the mean intensity over the entire expression phase:

(3) $$I=\frac{1}{N}\sum_{i}^{N}{Val}_{i}$$

where $Val_{i}$ represents the intensity of the specific emotion (Happy/Sad) at time i derived from FBSs. Duration ($T_{happy}$,${\;T}_{sad}$) is calculated as the total time the emotional intensity exceeded a threshold θ:

(4) $$T=∆t\cdot \sum_{i}1\left({Val}_{i}>\theta \right)$$

where Δt is the sampling interval and $1\left(\cdot \right)$ is the indicator function, and θ is set to 0.1. Latency ($L_{happy}$, $L_{sad}$) is defined as the time from instruction onset ($t_{0}$) to the moment intensity first exceeds θ:

(5) $$L\;=\;min\left\{(t_{i}-t_{0})\;|\;{Val}_{i}\;>\theta \right\}$$

if the threshold was not exceeded, latency was recorded as 5 s. These metrics quantify the intensity, persistence, and dynamic onset of emotional expression.

MCT (2-dim): This task evaluates the interplay between working memory and dynamic attention. We extracted attention allocation ability ($A_{a}$) from the search phase and calculation latency ($L_{c}$) from the calculation phase. Specifically, $A_{a}$ is defined as $T_{c}/T_{all}$, where $T_{c}$ is the total fixation time on the correct target and $T_{all}$ is the total search phase duration (5 s); a higher value indicates effective attention focusing. $L_{c}$ is defined as $T_{f}$ − $T_{0}$, where $T_{f}$ is the time of the first fixation on the correct option during the calculation phase, and $T_{0}$ is the onset time of the options. The final value for each metric is the average of two trials. We use Unity’s ray collision effects to detect whether the subject’s gaze is focused on the target object.

ST (8-dim): Reflecting oculomotor control and inhibitory executive functions, we extracted eight features from the AS and PS tasks based on [39,40]. These features include latency ($L_{s}$), mean velocity ($V_{m}$), peak velocity ($V_{max}$), and amplitude ($A_{s}$) for both task types. Before feature extraction, we excluded anticipatory saccades with latencies of less than 100 ms according to the method described in [40]. Following this, we sequentially extracted features from the processed data and averaged the final metrics across all trials. These metrics are critical for identifying deficits in saccadic initiation and suppression.

#### 2.5.3. Classification Model

**Figure 5:**
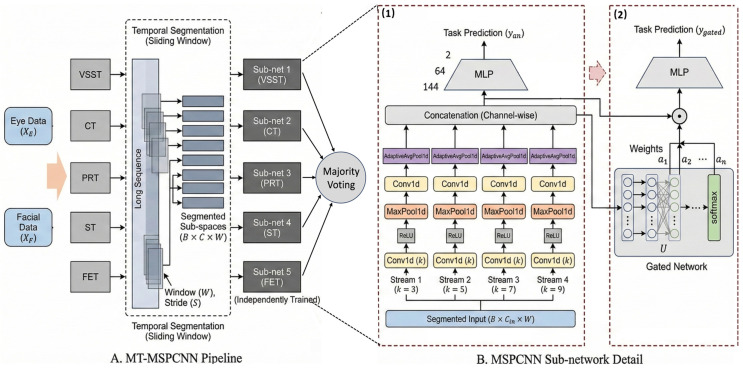
Flowchart of data preprocessing and the training process. (**1**) First phase: training the temporal feature extraction network. (**2**) Second phase: final classification using MIL.

In behavioral data analysis, effectively mining deep patterns in time-series data is key to improving classification performance. Given the aforementioned applicability of CNNs to eye movement and facial expression data, this paper proposes a multi-task multi-stream parallel convolutional neural network (MT-MSPCNN) to classify the HCs and the CD, the specific architecture of which is shown in Figure 5.

Specifically, we employed a classical CNN architecture to capture the latent information within the data. Given the temporal variability of the data in this study, the single convolutional kernels utilized in CNN are insufficient for a comprehensive representation. To address this, we designed a multi-scale parallel convolution module. By constructing multiple receptive fields with varying kernel sizes, this module aims to capture subtle features across a more comprehensive temporal scale. Furthermore, due to the long-term sequential nature of the data, analyzing the entire sequence directly can easily obscure critical local information. Therefore, we partitioned the complete sequences into multiple fixed-length slices and introduced Multiple Instance Learning (MIL) alongside an attention mechanism to effectively process the long time-series data in a weakly supervised manner. Finally, considering the inherent heterogeneity across different paradigms, we trained models independently for each task and employed a majority voting strategy to fuse the results for the final classification. This strategy is designed to leverage complementary information across tasks, thereby enhancing the diagnostic robustness. The specific implementation details are presented below.

In this study, the behavioral sequences exhibited by subjects typically demonstrate non-stationarity. Particularly for CD patients, discriminative data segments are often sparse and confined to specific regions. Consequently, we performed sub-space segmentation on the complete input sequences to achieve fine-grained feature extraction. Specifically, the preprocessed data from Section 2.5.1 were Z-score-standardized to serve as the model input. Let $X\in R^{C\times L}$ denote processed data, where C represents the number of feature channels and L denotes the total sequence length. Subsequently, a sliding window strategy was employed to decompose X into a set of N overlapping local sub-spaces $X=\{x_{n}{\}}_{n=1}^{N}$. The n-th temporal sub-space $x_{n}\in R^{C\times W}$ is defined as a subset of X:

(6) $$x_{n}={\left[v_{t}\right]}_{t\in T_{n}},T_{n}=t\in Z|\left(n-1\right)S<t\leq \left(n-1\right)S+W$$

where $v_{t}$ is the feature vector at time step t, W is the window size, and S is the stride. In this study, we set W=150 and S=50 to ensure sufficient temporal overlap, thereby preserving contextual continuity. Data segments at the tail end that were shorter than a full window were directly discarded. Additionally, the first four tasks in this study solely utilized eye-tracking data as input, whereas the FET employed a mixed input of eye-tracking and facial data.

In supervised model training, proper data annotation is crucial. Given the complex dynamics of the data in this study, assigning the global subject label to all sub-spaces inevitably introduces label noise. To eliminate this ambiguity and effectively mine the multi-scale patterns of the data, this study formulates it as an MIL task. Specifically, we employ a two-phase training strategy.

In the first phase, we train the MSPCNN to acquire foundational capability in capturing localized dynamics. Specifically, as illustrated in Figure 5(B1), the subject’s sub-space data $x_{n}$ is first fed into four parallel convolutional streams (with kernel sizes $s_{k}\in 3,5,7,9$) to capture local features across different scales. The features extracted by each stream are fused via channel-wise concatenation to form a comprehensive representation $z_{n}$, which is subsequently passed through an MLP head to output the predicted probability ${\hat{y}}_{n}$. Regarding label assignment, this study directly assigns the global subject label to all of its constituent sub-spaces. Studies have demonstrated that deep neural networks prioritize learning clean, simple patterns during training before memorizing noisy data [41]. Consequently, although hard-label assignment introduces noise, the model exhibits lower prediction loss on clean sub-spaces and higher loss on noisy sub-spaces during the early stages of training. To address this, we introduce a soft weighting mechanism that dynamically reduces the weights of high-loss samples via a decay function $w_{i}=exp(-\gamma \cdot {loss}_{i})$, thereby mitigating the negative impact of noise. Simultaneously, the number of training epochs is restricted to prevent the model from overfitting to noisy data in later stages. This strategy aims to balance the model’s representation capacity for localized dynamics with its robustness against label noise.

In the second phase, as illustrated in Figure 5(B2), the parameters of the pre-trained feature extractor are frozen to ensure robust multi-scale feature input. The attention mechanism and final classifier head are then optimized at the bag level using a batch size of 1. To dynamically resolve the labeling ambiguity, a Gated Attention mechanism [42] is introduced to assign weights to instance-level features. For the feature $z_{n}$, two parallel branches regulate information flow. The un-normalized attention score $e_{n}$ is calculated as:

(7) $$e_{n}=w^{⊤}\left(tanh\left(W_{V}z_{n}\right)⊙sigmoid\left(W_{U}z_{n}\right)\right)$$

where $W_{V}$, $W_{U}\in R^{K\times D}$ and $w\in R^{K\times 1}$ are learnable parameters; ⊙ denotes element-wise multiplication; D = 144; and K = 64. The normalized attention weight $a_{n}$ is obtained via softmax across all instances within the bag. The instances are then aggregated into a global bag-level representation $Z_{bag}$. Finally, the diagnostic is output using $Softmax\left(W_{c}Z_{bag}\right)$. The network is trained by minimizing the binary cross-entropy loss function.

(8) $$\left\{\begin{array}{c} a_{n}=\frac{exp\left(e_{n}\right)}{\sum_{j=1}^{n}exp\left(e_{j}\right)}\\ Z_{bag}=\sum_{n=1}^{N}a_{n}z_{n} \end{array}\right.$$

For each task, we trained a separate classification network using the same approach. Given the differences between tasks, we employ a majority voting strategy to integrate the prediction results from each task model. Let M=VSST,CT,PRT,ST,FET be the set of tasks. The final subject-level diagnosis ${\hat{Y}}_{subj}$ is determined by:

(9) $${\hat{Y}}_{subj}=arg\underset{c\in 0,1}{max}\sum_{m\in M}I\left({\hat{Y}}_{m}=c\right)$$

where ${\hat{Y}}_{m}$ is the predicted class from task m, and $I\left(\cdot \right)$ is the indicator function which equals 1 if the condition is true and 0 otherwise. This ensemble approach mitigates the variance of single-task predictions and enhances diagnostic robustness.

To evaluate the performance of MT-MSPCNN, several ML models were constructed for comparison. The training of the ML model utilized a total of 40 features, including the 27 features from Section 2.5.2, demographic characteristics, and 10 granular features from the MoCA-B. For the training phase, 15 CD and 15 HC subjects were randomly selected from the total cohort and divided into training and validation sets at a 7:3 ratio. The remaining 5 CD and 5 HC subjects served as an independent test set. To reduce randomness, training and evaluation were repeated 10 times, and the mean and standard deviation of the evaluation metrics were reported. Model performance was comprehensively evaluated using accuracy, sensitivity, precision, and F1-score. Specific training details are provided in Table 3. The proposed model was implemented in Python 3.9.12; ML models were based on scikit-learn 1.7.0, and MT-MSPCNN was implemented using PyTorch 2.8.0.

## 3. Results

### 3.1. Classification Performance

**Figure 6:**
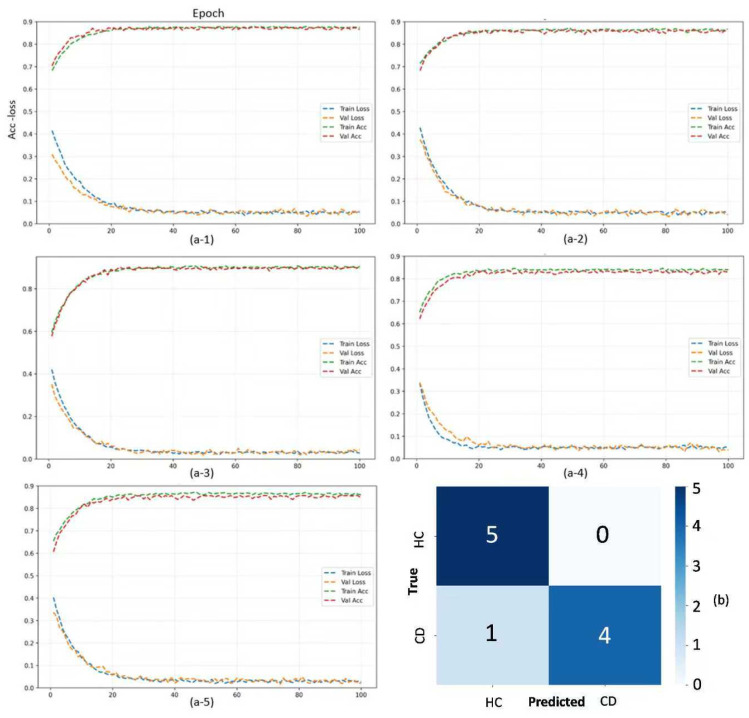
Training performance and classification results of the proposed MT-MSPCNN model. Training and validation curves (accuracy and loss) for the five task-specific subnetworks: (**a-1**) VSST, (**a-2**) MCT, (**a-3**) PRT, (**a-4**) ST, and (**a-5**) FET. All subnetworks show stable convergence. (**b**) Confusion matrix of the integrated MT-MSPCNN model on the independent test set, achieving an overall accuracy of 90%.

In this section, we will present the classification performance of MT-MSPCNN. To validate the effectiveness of deep feature extraction and compare the discriminative power across different tasks, this study trained five single-task subnetworks based on the aforementioned training scheme. Table 4 shows the average classification performance of each model during the training phase. Figure 6 shows the training curve of the optimal model and its classification performance on the test set.

The results indicate that the single-task model based on CNN outperforms the ML model across all metrics. Among these, GNB achieved the best classification performance in the ML model, attaining the highest Recall value. However, its lower Precision suggests a higher rate of misdiagnosis. In contrast, the single-task MSPCNN networks demonstrate higher stability across Accuracy, Precision, Recall, and F1-score metrics. Notably, among all single tasks, models trained using the PRT paradigm demonstrated the strongest discriminative power. This suggests that the physiological response-based paradigm, primarily regulated by the autonomic nervous system, is less susceptible to interference from task motivation or fatigue effects compared to other paradigms reliant on subjective participant cooperation. Consequently, it provides more objective and stable pathological features.

Furthermore, to evaluate the generalization capability of DL models on unseen sub-jects, we validated the overall performance of MT-MSPCNN using the reserved independent samples as the test set. At this stage, we achieved the final classification by aggregating the prediction results from each single-task model and performing voting. Table 5 presents the model’s average performance on the test set. ML models achieved accuracy ranging from 0.82 to 0.86, while MT-MSPCNN demonstrated significantly improved overall performance with an accuracy of 90%, as shown in the confusion matrix in Figure 6b. This result outperforms the best single-task model, suggesting that decision fusion strategies may offer potential advantages in mitigating individual heterogeneity fluctuations inherent in single-task approaches, thereby enhancing the robustness of auxiliary diagnosis.

### 3.2. Feature Statistical Results

**Figure 7:**
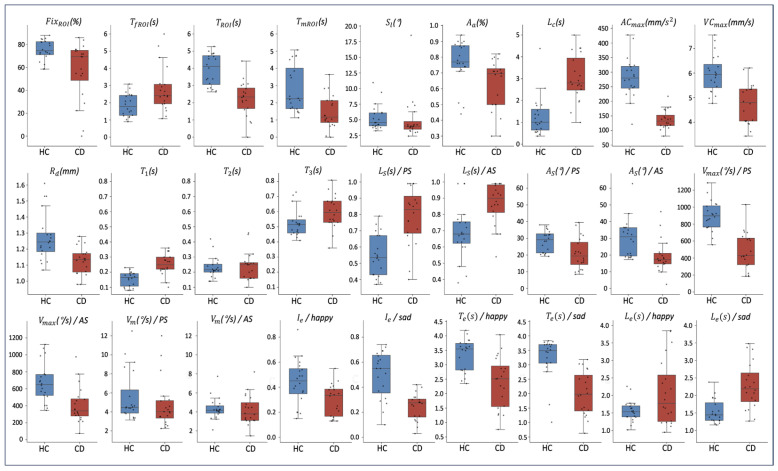
Box plots showing the distribution of 27-dimensional eye and facial expression features across participants. HC: healthy control; CD: cognitive decline.

**Figure A1:**
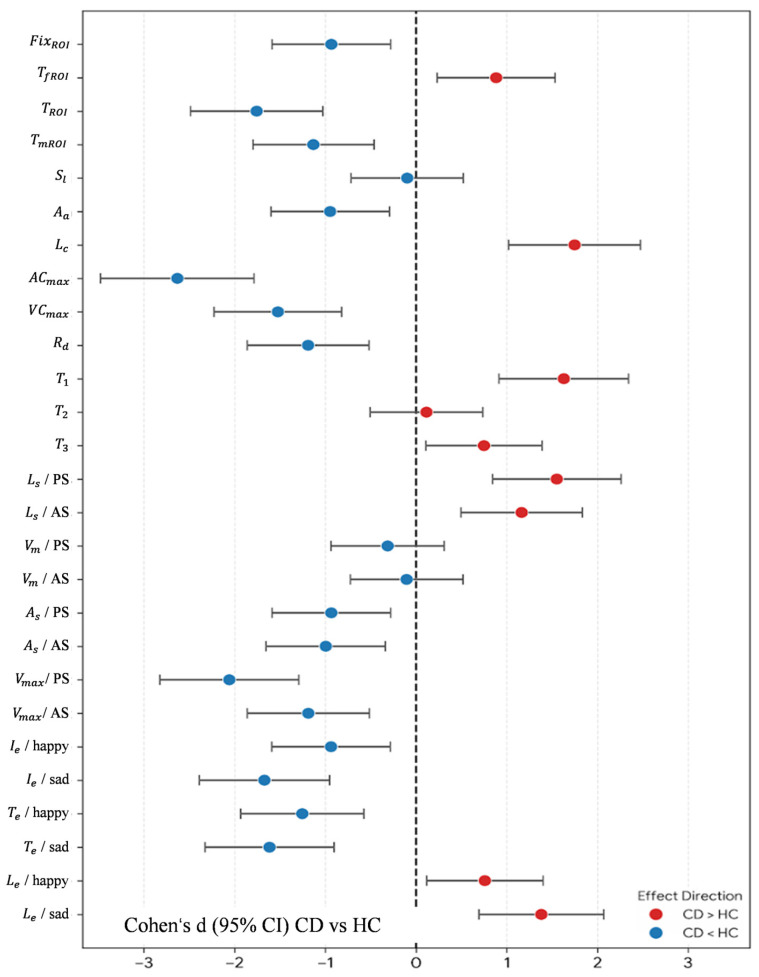
Effect size analysis of behavioral features between the healthy control (HC) group and the cognitive decline (CD) group across cognitive tasks. Positive effect sizes indicate higher values in the CD group relative to the HC group, and negative effect sizes indicate lower values. Effect sizes are reported as Cohen’s ‘d’ with 95% confidence intervals.

This study employed the Mann–Whitney U test to quantify statistical differences between the HC and CD groups across task-specific features. Table A1 and Figure A1 present the between-group feature differences and specificity analysis (Please refer to the Appendix A), while Figure 7 presents box plots illustrating the distribution of these features between groups. Overall, the CD group demonstrated pronounced degenerative patterns across all five tasks.

In the VSST, the CD group exhibited a marked decline in visual search abilities and difficulties sustaining attention. Specifically, patients showed a significant reduction in the percentage of fixations within the ROI (*p* = 0.01) and a significantly prolonged latency to first fixation on the ROI (*p* = 0.02). Furthermore, a significant decrease in both total fixation duration and median fixation duration (*p* < 0.01) reflected insufficient fixation stability on target objects.

For the MCT, the attentional allocation ability was lower in the CD group than in the HC group (*p* = 0.01), and the calculation latency was significantly increased (*p* < 0.01). This indicates that when cognitive load increases, individuals with CD exhibit underlying deficits in multitasking and information processing speed.

In PRT, the CD group exhibited delayed and attenuated pupillary dynamic regulation characteristics. Beyond increased pupillary response latency (*p* < 0.01) and prolonged time to maximum diameter change (*p* < 0.01), the maximum acceleration, maximum velocity, and diameter range of pupillary constriction were all significantly smaller than in the HC group (*p* < 0.01). This overall dynamic response delay suggests potential autonomic nervous system dysfunction.

For the ST, the CD group exhibited significantly longer mean saccade latency under both PS and AS conditions compared to the HC group (*p* < 0.01, *p* = 0.01), and a lower peak saccade velocity (*p* < 0.01). Notably, under the AS condition demanding higher cognitive control, the CD group exhibited a significantly reduced average amplitude (*p* = 0.01), reflecting to some extent the early decline in patients’ inhibitory control function.

Finally, in the FET, the CD group exhibited a behavioral pattern of “emotional blunting.” Regardless of whether expressing joy or sadness, patients demonstrated significantly reduced expression intensity (*p* < 0.01), shorter duration (*p* < 0.01), and markedly prolonged activation latency (*p* < 0.01). These characteristics suggest impaired control over facial muscle movements and disrupted emotional response circuits in patients with cognitive impairment.

## 4. Discussion

This study constructed five cognitive assessment paradigms based on VR, systematically collected eye movement and facial expression data from 40 participants and developed an MT-MSPCNN classification model. The model achieved 90% accuracy in distinguishing between HCs and CD, outperforming machine learning methods (82–86%). Furthermore, through statistical analysis, we identified a set of eye movement and facial features associated with cognitive decline. Based on these findings, this section delves into the significance of these behavioral features, analyzes the performance of the proposed model, and discusses the limitations of this study as well as potential directions for future research.

### 4.1. Model Training and Validation

Regarding classification models, the MT-MSPCNN constructed in this study effectively distinguishes between HCs and CD, demonstrating the effectiveness and generalization capability of the CNN architecture in processing time-series data.

As shown in Figure 6a, the sub-networks for each task exhibit stable convergence trends without severe oscillations. The smooth decline in the loss curves indicates that the multi-stream architecture can effectively optimize and fit the high-dimensional temporal patterns inherent in the eye-tracking and facial expression signals. This stable optimization trajectory may be attributable to the strategy adopted during the pre-training phase, which mitigates the risk of rapid overfitting under limited data conditions to some extent while maintaining training stability.

Finally, the confusion matrix (Figure 6b) details the performance on the independent test set. Although the test cohort is small in scale, the integrated MT-MSPCNN correctly classifies 9 out of 10 unseen subjects, providing preliminary validation of its predictive capability on novel data.

### 4.2. Behavioral Features

Compared to previous studies, the feature results extracted in this research demonstrate both consistency and expansion in multiple aspects. In the VSST, CD patients generally exhibited a lower proportion of fixation within the ROI and a significantly delayed time to first entry into the ROI, a trend consistent with findings in the literature [15]. Furthermore, we observed that the median fixation duration within the ROI was significantly lower in the CD group than in the HC group, suggesting a diminished ability to sustain visual attention. Notably, this finding also aligns with conclusions from earlier studies regarding fixation patterns and cognitive decline [43].

For the MCT, the CD group showed a significant decrease in attention directed toward the target during the search phase and a significantly prolonged response latency during the calculation phase. This may indicate that CD patients are unable to effectively focus their attention on target stimuli, suggesting deficits in attention allocation and short-term memory processing. It should be noted that this study is limited by the elderly population’s acceptance of VR technology, resulting in a small initial sample size with a certain degree of heterogeneity; therefore, the current results remain at a preliminary validation stage. Future research will expand the sample size through multi-center collaborations to further validate the unique patterns revealed by this dynamic paradigm.

In the PRT, the CD group exhibited pupillary dynamic characteristics distinct from the HC group, specifically manifested as significant reductions in diameter range, maximum change velocity, and maximum acceleration, along with significant prolongations in pupillary response latency and time to maximum diameter change. This trend is consistent with reports on AD patients in the literature [38], suggesting a possible underlying mechanism involving central cholinergic dysfunction.

In the ST, the CD group exhibited longer saccade initiation latency, lower peak saccade velocity, and reduced saccade amplitude, consistent with results reported in the literature [39,40]. This reveals dysfunction within the oculomotor system in the cognitively impaired population. On the other hand, the mean saccade velocity showed no significant inter-group difference in our study, a finding that may be related to the internal heterogeneity of the CD group. It should be noted that our study divided the groups based solely on total scale scores without further evaluating disease severity; therefore, we are temporarily unable to quantify the specific impact of this heterogeneity on individual metrics. Future research will incorporate more detailed analyses of disease severity within larger-scale datasets to clarify the differential patterns of oculomotor metrics between MCI and AD.

Finally, in the FET, this study found that both facial expression intensity and duration were significantly reduced in the CD group, consistent with the phenomenon of generally diminished emotional expression reported in patients with neurodegenerative diseases in the literature [27]. Furthermore, we observed a significantly prolonged expression onset latency in the CD group, suggesting that CD patients may not only have deficits in the maintenance of expressions but also exhibit specific impairments in the initiation process of the emotional motor pathway. Given the current sample size, these findings are preliminary observations and require further confirmation in larger-scale studies. It is noteworthy that the FT sensor used in this study, beyond recognizing basic emotions like happiness and sadness, is capable of synchronously capturing fine-grained parameters of various FBSs, including lip movements, jaw movements, and brow furrowing. Therefore, future research could move beyond basic emotions by integrating these more comprehensive and granular facial features to explore the state of neurocognitive function.

### 4.3. Limitations and Future Work

This study still faces several challenges and limitations during its advancement. First, the dataset relied upon for model construction and validation originates from a single center and has a limited sample size. Although subspace exploration was employed during model training to enhance data diversity, its generalization capabilities still require further validation in larger-scale, multi-center populations. Second, this study employs a cross-sectional design, making it difficult to capture the dynamic progression of the disease. Furthermore, while combining MCI and AD into CD enhances statistical robustness, the internal class imbalance within this group might introduce a potential risk of model bias, obscuring subtle differences between the disease stages. At the methodological level, our research on the impact of hyperparameters (e.g., window size for subspace partitioning) remains insufficient. To address these limitations, future research will focus on several key directions. First, we aim to validate the model’s robustness and generalization capabilities within larger-scale, multicenter longitudinal cohorts. Second, we will systematically investigate the impact of hyperparameter configurations on model performance. Meanwhile, more detailed assessments of disease severity will be incorporated to quantify the differences in features across different stages of the disease course. Furthermore, we plan to incorporate additional data modalities—such as EEG, neuroimaging, and linguistic text to explore more advanced multimodal fusion architectures. This will be coupled with comprehensive ablation studies to explicitly evaluate the distinct contributions of individual cognitive tasks and specific modalities. Concurrently, we will integrate advanced interpretability techniques to enable the precise localization of key discriminative features and facilitate the root-cause analysis of abnormal behavioral patterns. Ultimately, these efforts will accelerate the translation of our proposed model into a viable clinical application.

## 5. Conclusions

Digital biomarkers based on eye and facial expressions are gaining increasing attention in the field of AD detection. However, most existing studies focus on single tasks and feature-engineering approaches, leaving deep learning-based multimodal fusion and ecologically valid experimental settings underexplored. To address these limitations, this study developed five cognitive paradigms based on VR and systematically collected time-series data on eye movements and facial expressions. Based on this, this study proposed the MT-MSPCNN architecture. By utilizing four parallel convolutional branches, this model achieved effective integration of cross-task and cross-modal features, obtaining a preliminary accuracy of 90% in distinguishing between the CD and HC groups. Its performance significantly surpassed that of ML methods and single-task models. Furthermore, this study validated the effectiveness of several known cognition-related features across multiple tasks and explored a series of potential behavioral feature patterns, providing novel perspectives for the early screening of cognitive impairment.

## Figures and Tables

**Table 1:** Basic characteristics of the subjects.

Variable	HCs (*n* = 20)	CD (*n* = 20)	*p* Value
Age (years), Mean ± std	70.55 ± 9.01	77.35 ± 11.17	0.07
Female, *n* (%)	13 (65%)	10 (50%)	0.33
Education (years), Mean ± std	9.45 ± 1.76	8.85 ± 2.06	0.24
MoCA-B (score), Mean ± std	25.20 ± 1.32	18.35 ± 4.48	<0.01

**Table 2:** Explanation of the feature definitions of eye movement and facial expressions. (Num: Number of features).

Task	Feature	Description	Num
VSST	${Fix}_{ROI}$ (%)	The proportion of the total fixation time when the gaze point is on ROI	1
	$T_{fROI}$ (s)	The time difference between the start time of the search stage and the time spent on the first gaze ROI	1
	$T_{ROI}$ (s)	Total dwell time (including both fixations and intra-ROI saccades) within the search stage	1
	$S_{l}$ (°)	The median of all saccade distances during the search stage	1
	$T_{mROI}$ (s)	The median fixation time within ROI	1
MCT	$A_{a}$ (%)	The proportion of time allocated to all correct targets during the search phase to the total time of the search phase	1
	$L_{c}$ (s)	The time when the correct answer is first discovered during the calculation stage	1
PRT	${AC}_{max}$ (mm/$s^{2}$)	The peak of the acceleration during the pupil contraction process	1
	${VC}_{max}$ (mm/s)	The peak speed during the pupil contraction process	1
	$R_{d}$ (mm)	The difference between the maximum and minimum diameters of the pupil during the stimulation period	1
	$T_{1}$ (s)	The time interval from the start of light stimulation to the occurrence of a detectable contraction response in the pupil	1
	$T_{2}$ (s)	The time elapsed from the moment the pupil begins to contract until its contraction speed reaches its peak	1
	$T_{3}$ (s)	The time elapsed from the moment the pupil begins to contract until it reaches its maximum degree of contraction	1
ST	$L_{s}$ (s)	The average time interval from the appearance of the target to the start of saccade (PS & AS)	2
	$V_{m}$ (°/s)	The average speed of all effective saccades (PS & AS)	2
	$V_{max}$ (°/s)	The peak value of the recorded saccade velocity among all effective saccades (PS & AS)	2
	$A_{s}$ (°)	The average amplitude of all effective saccades (PS & AS)	2
FET	$I_{e}$	Mean facial expression intensity during the expression period (happy & sad)	2
	$T_{e}$ (s)	Maximum sustained duration of the expression per trial (happy & sad)	2
	$L_{e}$ (s)	Time from task cue onset to the first significant change in expression intensity (happy & sad)	2
Total feature			27

**Table 3:** Summary of ML & DL classifiers employed in this study.

Model	Information	
LR	L2 penalty, regularization rate = 1.0	
GNB	—	
SVM	RBF kernel, γ = “scale,” grid search on C = 0.1, 0.4, 0.7, 1	
MT-MSPCNN	Adam optimizer, lr = 0.001, Cross-entropy loss function	
	Stream1	Conv1D (*c*, 16, *k* = 3, *p* = 1); Maxpool1d (*k* = 2); Conv1D (16, 32, *k* = 3, *p* = 1), AdaptiveAvgPool1d (1)
	Stream2	Conv1D (*c*, 32, *k* = 5, *p* = 2); Maxpool1d (*k* = 4); Conv1D (32, 64, *k* = 5, *p* = 2), AdaptiveAvgPool1d (1)
	Stream3	Conv1D (*c*, 8, *k* = 7, *p* = 3); Maxpool1d (*k* = 2); Conv1D (8, 16, *k* = 3, *p* = 1), AdaptiveAvgPool1d (1)
	Stream4	Conv1D (*c*, 16, *k* = 9, *p* = 4); Maxpool1d (*k* = 3); Conv1D (16, 32, *k* = 5, *p* = 2), AdaptiveAvgPool1d (1)

**Table 4:** Classification performance comparison between ML and DL models. Mean ± std. Bold font indicates the best performance.

Model	Group (HCs vs. CD)			
	Accuracy	Precision	Recall	F1-Score
GNB	0.84 ± 0.045	0.79 ± 0.114	**0.87 ± 0.021**	0.83 ± 0.059
SVM	0.82 ± 0.023	0.81 ± 0.012	0.84 ± 0.024	0.82 ± 0.017
LR	0.81 ± 0.032	0.82 ± 0.033	0.82 ± 0.031	0.81 ± 0.012
VSST-MSPCNN	0.85 ± 0.025	0.85 ± 0.015	0.86 ± 0.017	0.84 ± 0.021
MCT-MSPCNN	0.84 ± 0.023	0.83 ± 0.021	0.85 ± 0.015	0.83 ± 0.022
PRT-MSPCNN	**0.88 ± 0.021**	**0.86 ± 0.033**	0.85 ± 0.013	**0.86 ± 0.013**
ST-MSPCNN	0.81 ± 0.025	0.83 ± 0.022	0.83 ± 0.021	0.83 ± 0.015
FET-MSPCNN	0.84 ± 0.017	0.84 ± 0.018	0.84 ± 0.023	0.84 ± 0.014

**Table 5:** Model performance averaged across 10 random test sets. Bold font indicates the best performance.

Model	Group (HCs vs. CD)			
	Accuracy	Precision	Recall	F1-Score
NB	0.85	0.80	0.94	0.86
SVM	0.82	0.83	0.84	0.83
LR	0.86	0.82	0.92	0.87
MT-MSPCNN	**0.90**	**0.83**	**1.00**	**0.91**

## Data Availability

Given the sensitivity of the data and to comply with the GDPR and institutional ethical guidelines, this dataset is not publicly available. Data access may be requested from the corresponding author upon reasonable request and subject to obtaining additional ethical approval.

## Appendix A

**Table A1:** Comparison of behavioral features between HC and CD groups across cognitive tasks.

Task	Feature	HC (Mean ± Std)	CD (Mean ± Std)	*p*-Value
VSST	${Fix}_{ROI}$ (%)	75.29 (8.79)	57.30 (25.06)	0.01
	$T_{fROI}$ (s)	1.86 (0.65)	2.68 (1.28)	0.02
	$T_{ROI}$ (s)	3.93 (0.90)	2.23 (1.01)	<0.01
	$T_{mROI}$ (s)	2.81 (1.32)	1.47 (0.94)	<0.01
	$S_{l}$ (°)	5.36 (1.97)	5.08 (3.42)	0.09
CT	$A_{a}$ (%)	0.76 (0.14)	0.62 (0.16)	0.01
	$L_{c}\;(s)$	1.28 (0.90)	3.03 (1.04)	<0.01
PRT	${AC}_{max}$ (mm/$s^{2}$)	282.46 (68.94)	137.60 (31.69)	<0.01
	${VC}_{max}$ (mm/s)	5.98 (0.75)	4.63 (0.81)	<0.01
	$R_{d}$ (mm)	1.27 (0.15)	1.13 (0.08)	<0.01
	$T_{1}$ (s)	0.15 (0.05)	0.25 (0.07)	<0.01
	$T_{2}$ (s)	0.23 (0.07)	0.24 (0.09)	0.77
	$T_{3}$ (s)	0.52 (0.09)	0.60 (0.10)	0.02
ST	$L_{s}$ (s)/PS	0.55(0.12)	0.79 (0.17)	<0.01
	$L_{s}$ (s)/AS	0.71 (0.16)	0.85 (0.12)	0.01
	$V_{m}$ (°/s)/PS	5.57 (2.61)	4.75 (2.52)	0.16
	$V_{m}$ (°/s)/AS	4.33 (1.12)	4.19 (1.51)	0.44
	$A_{s}$ (°)/PS	28.40 (6.20)	20.90 (9.17)	0.01
	$A_{s}$ (°)/AS	30.05 (11.17)	19.45 (9.54)	0.01
	$V_{max}$ (°/s)/PS	898.47 (172.99)	483.27 (217.55)	<0.01
	$V_{max}$ (°/s)/AS	673.69 (227.36)	407.22 (209.68)	<0.01
FET	$I_{e}$/happy	0.44 (0.18)	0.30 (0.12)	<0.01
	$I_{e}$/sad	0.50 (0.18)	0.24 (0.10)	<0.01
	$T_{e}$ (s)/happy	3.33 (0.55)	2.38 (0.88)	<0.01
	$T_{e}$ (s)/sad	3.23 (0.72)	2.02 (0.74)	<0.01
	$L_{e}$ (s)/happy	1.55 (0.30)	2.08 (0.92)	<0.01
	$L_{e}$ (s)/sad	1.54 (0.33)	2.28 (0.65)	<0.01
